# Author Correction: Quantum capacities of transducers

**DOI:** 10.1038/s41467-025-60651-2

**Published:** 2025-06-20

**Authors:** Chiao-Hsuan Wang, Fangxin Li, Liang Jiang

**Affiliations:** 1 Department of Physics and Center for Theoretical Physics, National Taiwan University, Taipei, 10617 Taiwan; 2 Center for Quantum Science and Engineering, National Taiwan University, Taipei, 10617 Taiwan; 3 Physics Division, National Center for Theoretical Sciences, Taipei, 10617 Taiwan; 4 Pritzker School of Molecular Engineering, University of Chicago, Chicago, IL 60637 USA

**Keywords:** Quantum information, Quantum optics, Information theory and computation, Fibre optics and optical communications

Correction to: *Nature Communications* 10.1038/s41467-022-34373-8, published online 05 November 2022

The original version of this article contained an error in the unit convention for continuous-time quantum capacities, which should be rescaled by a factor of 1/(2π) to reflect frequency rather than angular frequency. This affected equations (3), (5), (12), (13), (14), (15), (29), (35) and (37), and Figures 2, 3, 4, 6, 7, 8, and 9. The resulting value for the maximal qubit communication rate of a transducer ‘*Q*^max^’ reported in the Abstract, Introduction, Results (below Eq. (14)), and Discussion, should be equal to ‘5 *g*_max_’ instead of ‘31.4 *g*_max_’. All affected equations and figures have been corrected accordingly in the PDF and HTML versions of the article.

